# Integrated Edge Deployable Fault Diagnostic Algorithm for the Internet of Things (IoT): A Methane Sensing Application

**DOI:** 10.3390/s23146266

**Published:** 2023-07-10

**Authors:** S. Vishnu Kumar, G. Aloy Anuja Mary, Miroslav Mahdal

**Affiliations:** 1 Department of Electronics and Communication Engineering, Vel Tech Rangarajan Dr. Sagunthala R&D Institute of Science and Technology, Avadi 600062, India; mail2jitvishnu@gmail.com (S.V.K.); aloyanujamary@gmail.com (G.A.A.M.); 2 Department of Control Systems and Instrumentation, Faculty of Mechanical Engineering, VSB-Technical University of Ostrava, 17. Listopadu 2172/15, 70800 Ostrava, Czech Republic

**Keywords:** sensor faults, Sensing Edge Device, edge fault detection, Random Forest, Fault Tree Analysis, Methane Sensing

## Abstract

The Internet of Things (IoT) is seen as the most viable solution for real-time monitoring applications. But the faults occurring at the perception layer are prone to misleading the data driven system and consume higher bandwidth and power. Thus, the goal of this effort is to provide an edge deployable sensor-fault detection and identification algorithm to reduce the detection, identification, and repair time, save network bandwidth and decrease the computational stress over the Cloud. Towards this, an integrated algorithm is formulated to detect fault at source and to identify the root cause element(s), based on Random Forest (RF) and Fault Tree Analysis (FTA). The RF classifier is employed to detect the fault, while the FTA is utilized to identify the source. A Methane (CH4) sensing application is used as a case-study to test the proposed system in practice. We used data from a healthy CH4 sensing node, which was injected with different forms of faults, such as sensor module faults, processor module faults and communication module faults, to assess the proposed model’s performance. The proposed integrated algorithm provides better algorithm-complexity, execution time and accuracy when compared to FTA or standalone classifiers such as RF, Support Vector Machine (SVM) or K-nearest Neighbor (KNN). Metrics such as Accuracy, True Positive Rate (TPR), Matthews Correlation Coefficient (MCC), False Negative Rate (FNR), Precision and F1-score are used to rank the proposed methodology. From the field experiment, RF produced 97.27% accuracy and outperformed both SVM and KNN. Also, the suggested integrated methodology’s experimental findings demonstrated a 27.73% reduced execution time with correct fault-source and less computational resource, compared to traditional FTA-detection methodology.

## 1. Introduction

**Figure 1:**
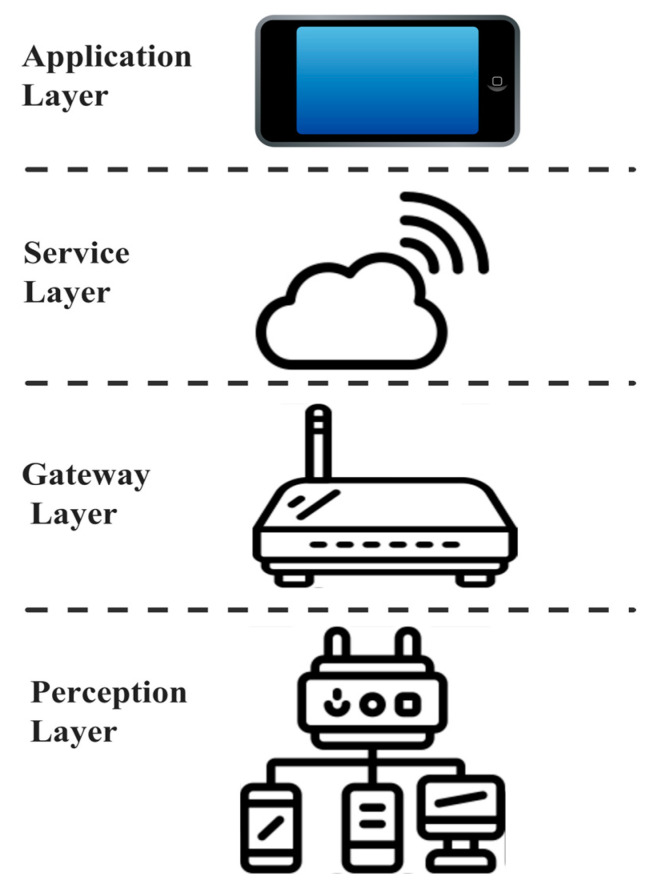
Key Building Blocks of an IoT Architecture.

The IoT is a network of physical objects that are rapidly being adapted into environmental monitoring for collecting, sharing and using the data for automation purpose through the Internet [1,2,3]. Based on advancements in fields such as microelectronics, sensing, communication, and distributed systems, it is a rapidly expanding area that is changing how humans interact with the physical world [4]. IoT devices have the ability to be remotely controlled, monitored, and optimized, which can increase productivity, reduce costs, and enable a variety of applications, such as environmental monitoring, healthcare, supply chain, and inventory management [5]. The general four-layer architecture of an IoT system [6] is illustrated in Figure 1. The perception layer is responsible for collecting the parameters of interest from the environment where the Sensing Edge Devices (SEDs) are deployed. The gateway layer is responsible for sharing/collecting information among SEDs and communicating it to the application layer through a service layer. The application layer is where the collected data from the perception layer is processed into meaningful information to direct the data-driven system or applications [7].

Despite being adapted into multiple functional areas, such as healthcare, agriculture, waste treatment and industrial automation, the IoT still face issues related to identifying faulty data being collected and shared [8,9]. Also, due to its heterogenous nature, the security and privacy of the architecture is also not standardized [10]. From the four-layer architecture it is understandable that the data collected from the environment has a greater impact over the entire IoT architecture, as any corruption in the SED-data can result in overall system failure [11]. SED faults occur for a variety of reasons, such as wear and tear [12], calibration error, physical damage, hostile environment due to heat [13], vibration [14], network failure or intentional tampering [15]. A SED may sustain the damage but then the fault must be identified as soon as it originates to save the data-driven system. In addition, the faulty element(s) must be recognized to make timely repair to increase the SED’s availability time. The other major reasons attributed to find faults in an IoT environment are the considerable amount of bandwidth and power that can be saved [16], which are really important in a resource constraint application such as remote gas sensing.

The conventional knowledge-driven, fault-finding approach involves data collection and the application of expertise; however, this approach remains inadequate to diagnose new failures, which can be replaced by data-driven techniques [17]. Physical inspections of SEDs can be challenging as they are frequently deployed in hard-to-reach isolated locations. Thus, remote diagnosis is preferred for fault identification, but the methodology suffers a major bottleneck—faulty connectivity emphasizing edge detection. Consequently, failure detection is a crucial operation and a fundamental requirement of all remotely deployable SEDs to increase the reliability and availability of an IoT system [18].

### 1.1. Motivation and Contribution

The main objective of this research is to detect, fault at its source with better accuracy and narrow down the faulty element(s). Using fault-detecting algorithm to identify the occurrence has been widely reported, but identifying the responsible element(s) is more important to reduce the repair time and to increase the availability. Thus, a comprehensive fault diagnostic architecture by integrating the classification tree and deductive tree is proposed. Care has been taken to develop the diagnostic algorithm to consume limited resource to facilitate edge deployment and to compress the Cloud’s stress.

### 1.2. Related Work

Faults are generally defined as a malfunction or deviation from a normal behavior of a system. In an IoT environment, faults may occur in any of the four-layers and may propagate to the other layer(s). From a perception layer perspective, once fault occurs, it requires repair time, affecting the SED’s availability [19]. The reported the literature has a wide range of faults, such as bias, drift, gain and accuracy degradation, but faults that occur in an IoT-SED is much greater [20]. Hard and soft faults are the two categories under which the SED failure can be classified; a soft fault is defined as transmitting an incorrect value, while any physical damage to SED resulting in erroneous value is termed as a hard fault [21]. The authors [22] have recognized that SED faults can be caused by a variety of issues, including calibration errors, hardware failures, hostile environments, or link failures.

In the literature, the recent works proposed by the authors [23] showcases a framework that applies Machine Learning (ML) models such as Decision Tree (DT) and Gaussian naïve Bayes to detect fault. The authors have greatly contributed by designing the algorithm to be edge deployable, but the proposed methodology covers only three types of faults, namely: Spike, Struct, and Bias fault. In addition, there is no reference to identify the element(s) responsible for the failure. The work [24] proposes an edge deployable, parameter transplantation-based, fault detection approach, which the authors developed and deployed in an embedded system to track the status of a bearing in real-time. However, the process has several drawbacks, like the requirement to gather enough tagged data each time a new model is acquired. The authors themselves have emphasized the need for additional study to lessen the volume of data required to train the model and cut costs. A new Deep Learning (DL)-based study was proposed by the authors [25], where an edge-AI and a specially created middleware solution based on FIWARE was used. The proposed architecture was subjected to tests, in an industrial autonomous transfer vehicle as a case study. It is apparent that this system has large hardware requirements and, additionally, the configuration necessitates the Robotic Operating System (ROS) for proper operation. This is inappropriate for resource-constrained applications such as remote gas monitoring with drones. The research work [26] offers an Echo State Networks model that takes advantage of sensor data fusion that extracts the dynamics from a collection of heterogeneous sensor data to detect fault. The authors orchestrated the edge device using cloud resources, and have not explicitly mentioned the minimal hardware requirements for real-time implementation, which is fundamentally relevant in terms of quick resource requirement and power consumption for application-like, drone-based gas sensing. In addition, when the number of sensors increases, the processing power and memory requirements for sensor fusion also increases. This results in a reduced real-time performance and increased latency. Additionally, for sensor data fusion, maintaining sensor compatibility depends heavily on sensor calibration.

The work [27] proposed the use of edge computing for fault diagnosis, in which the authors verified that ML models, such as DT, RF, and SVM, can be implemented over a low-cost, resource-constrained, edge computational board—Arduino Mega, which provided the necessary motivation for our work. Soft computing methods such as RF, K-NN and SVM have been effectively applied in an automatic fault detection system during the past few decades [28,29]. For the purpose of fault detection, each of these techniques has been effectively applied with modification, However, further research is required in identify the problematic element to reduce repair time. In contest to deploying the soft computing algorithm, many successful mechanisms have been proposed by reputed research groups, which are generally categorized as centralized, distributed and hybrid approaches [21,30]. Most of the proposed techniques are based on statistical methods, anomaly detection or based on ML. In a centralized approach, the algorithms are deployed in a central-node, which keeps track of each individual SEDs by diagnosing the trans-received message through algorithms such KNN [31]. But the bandwidth requirement and the cost associated with this is quite expensive. The drawbacks of centralized fault detection can be eliminated through a distributed approach [32], where ML algorithms such as SVM are implemented in a cluster head to identify the faulty SED. But this method suffers from the need of higher dataset. On the other hand, RF is a collective learning technique that finds applications in both classifications and regression [33] applications. The authors [34] proposed a hybrid algorithm based on feed-forward Neural Networks, but it does not identify the faulty element(s). The authors [35] also proposed the usage of a K-NN algorithm for fault identification, but this method suffers to capture the small changes, making them not suitable for instantly changing CH4 concentration. FTA is a deductive-graphical model that can be used in the design and operations phase. In the design phase, FTA is utilized to debug the design process, and in the operations phase, it can be used to identify the fault with the root cause of failure. When FTA is used for fault analysis, the general operational structure of the system is transformed into a structured logic tree diagram made up of logic gates and events [36]. The events reflect problematic conditions, and the gates represent the logical connections between the events [37]. The top event, which represents the overall system failure, is defined as the first step in the deductive process and the potential underlying root-causes are worked out backward. The work [11] shows the use of FTA and Fuzzy Neural Network in identifying sensor fault. An aquaculture application was used to demonstrate the merits of the proposed algorithm, but the work focused only on sensing faults, namely, constant deviation, biased sensor value, numerical mutation and supply voltage fault. In addition, the authors left a research gap with respect to identifying the element responsible for the fault to occur. The study [38] proposed the use of FTA for analyzing unexpected occurrences of events on C-written-embedded control software for industrial appliances. By identifying the root causes of a major occurrence and implementing preventative action, the suggested strategy achieves a secure ECSW. In the work [39], the authors suggested an FTA-based method for assessing the autonomous underwater vehicle’s state of health in real time. Performance, reliability, and defect information were employed as measures to assess the suggested architecture. As per the authors, as the size and complexity of the system rises, the FTA becomes more computationally expensive. To solve the shortcomings of fault identification in complex systems, the author [40] developed a system-phenomenon fault tree (SPF). The authors of work [41] contrasted fuzzy FTA with the traditional approach and demonstrated that different algorithms coupled with FTA can improve end results.

Knowledge-based techniques frequently mimic human intelligence by employing “if-then” principles. IoT technologies like Expert Systems (ES) are frequently used in these procedures. Although ES have been used to diagnose faults in many different fields, they struggle with knowledge acquisition and rule explosion, where there is a tendency for the systems to become more and more complicated. In these cases, multiple technologies are coupled to address these issues [42]. This motivated the current work, where RF and FTA are combined to detect the faults and the source of faults in an IoT-based gas sensing application.

The remaining sections of the article are organized as follows; Section 2 introduces SED as a system, and details the workings, associated faults, and briefs about the proposed integrated algorithm, and the place where it is implemented in a gas sensing architecture. Section 3 accounts the inference made from the experiment and Section 4 discusses the future aspects and conclusion of the work.

## 2. Materials and Methodology

A CH4 gas sensing application is used as the case study to verify the proposed integrated approach in edge fault finding. The sub-sections are arranged in following order, Section 2.1 describes in detail about the SED under study, Section 2.2 details about the integrated methodology and Section 2.3 explains the implementation of the proposed methodology.

### 2.1. Methane Sensing Edge Device

**Figure 2:**
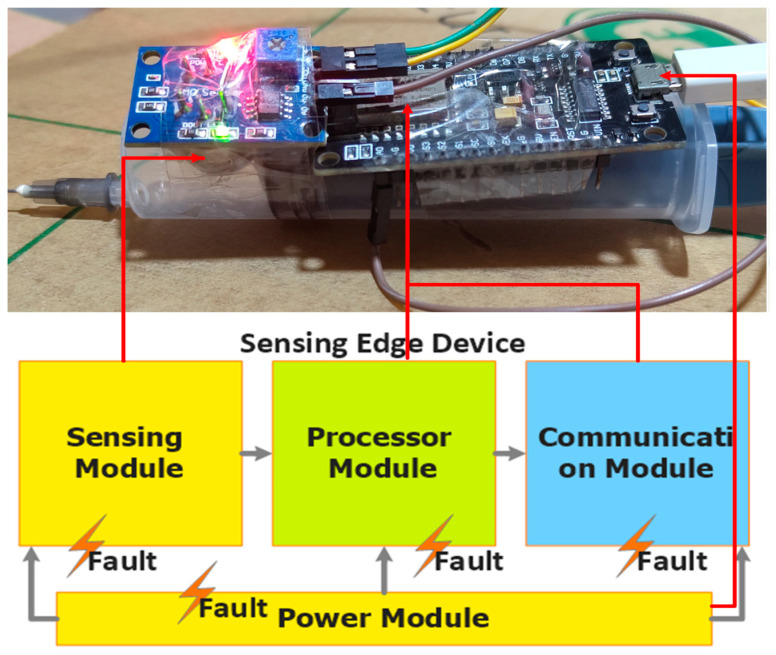
General Architecture of a Sensing Edge Device.

A SED is an incorporation of components, such as a sensor, microcontroller, communication and power module, as illustrated in Figure 2. It is deployed to collect the contextual information about the environment; in our case, the concentration of CH4 [43,44]. The SED under consideration is developed in-house, using commercially available components such as MQ-4, NodeMCU, 50 mL syringe, a USB-A- to Micro-B-type cable and a 5 V power bank.

#### 2.1.1. Sensing Module

The sensing module of an edge device is responsible for data acquisition. In the current study context, the MQ-4 sensor is used as a sensing module to sense the CH4 concentration. MQ-4 works by means of generating an equivalent electrical signal to the surrounding CH4 concentration, based on the reaction between the metal oxide semiconductor and the gas [45]. The electrical signal is then converted into parts per million values, programmatically. In the current study, the CH4 produced from coal-waste is measured between 200 ppm to 350 ppm and the same has been used as lower and upper threshold limits in the FTA model. If the SED is subjected to any physical damage, it could result in drawing more current due to short-circuiting or producing an inappropriate signal, which could also result in harming other peripherals connected with it, such as the microcontroller. Additionally, a harsh environment can also cause the sensing element to produce inaccurate signals or no signals, affecting the accuracy.

#### 2.1.2. Processor Module

The processor in the SED’s perspective is responsible for the sensed data and communicating it to the Cloud. In the current study of CH4 sensing, a Tensilica L106 chip is used. A damaged microcontroller could result in a situation where it is not able to process the sensor data or communicate the processed data to the outer world. To identify such situations, “User defined Error Codes” are introduced; an error code “E0” is sent to the Cloud, indicating a software issue happened and watchdog timer-initiated processor-reset. Similarly, error code “E1” is sent to the Cloud when the supply voltage reads below the threshold value, and “E2” is sent when the battery bank temperature is high (this functionality is not in the scope of the current work, but will be useful when the SED is used in a drone environment to sense the CH4 dispersion). Also, the processor is programmed to send an error code “E3” when the current sensor reads a sudden spike in current consumption; a sign of short-circuiting in an SED. When SEDs are deployed in a harsh environment, they are prone to temperature fluctuations, which could affect the sensor readings; thus, the on-chip temperature is also monitored and shared with the Cloud, and in the current study, the lower and upper threshold of the on-chip temperature has been limited between −40 degree Celsius and 125 degrees Celsius; in accordance with ESP8266′s datasheet.

#### 2.1.3. Communication Module

An individual communication module itself is a small processing device, and in the current study, an ESP8266 Wi-Fi chip is used. It is responsible for facilitating communications between the edge sensor and the cloud services. Any physical damage or fault in the software stack can result in a faulty communication. In the FTA, the Round-Trip Time (RTT), followed by a Link Failure event, becomes initiated when the base-station did not receive any payload value. In the current study, 12.32 ms is used as the RTT threshold value.

#### 2.1.4. Faults in SED

It is clear that the processor and communication module have dependencies. Thus, the probability of SED working as a single system can be calculated as the product of the probabilities of each component working while considering the dependencies, as in Equation (1), where p(S) represents a working sensor, p(P) represents a working processor and p(C) represents a working communication module. Similarly, the probability of the SED failing as a system can be calculated as in Equation (2), where q(S) represents a faulty sensor, q(P) represents a faulty processor and q(C) represents a faulty communication module.

P(SyW) = p(S) ×p(P|S) × p(C|P)(1)

P(SyF) = q(S) + [p(S) × q(P|S)] + [p(S) × p(P|S) × q(C|P)(2)

Equations (1) and (2) resemble the probability of the system having only two possible outcomes: either the system is working, or it is not working, thus promoting for a binary classification model.

### 2.2. Integrated Fault Diagnosis Algorithm

The proposed edge deployable fault detection algorithm is a combination of FTA with a binary classifier. FTA is a widely used methodology for identifying potential causes of system failures, However, it requires expert knowledge to be able to construct appropriately. Also, it consumes higher computational resources, which always becomes increased when new events and logics are added [39]. On the other hand, RF is a powerful classification algorithm to identify faulty sensor data, but fails to identify individual element(s) responsible for fault occurrence [46]. Thus, RF is used to invoke the FTA function on a case-to-case basis. This arrangement reduces the need for higher computational requirements in each cycle. This integration is particularly useful in resource constrained complex systems, where it may be difficult to identify the root causes of failures using traditional FTA. The following section provides a short brief on the technicality of FTA and RF models.

#### 2.2.1. Fault Tree Analysis

FTA is a graphical model with a tree-like structure that uses logic gates and events to represent problematic conditions and the connections between them. The system failure is represented by the top event, and the underlying root causes are determined backwards. FTA follows two approaches for assessing fault, namely: qualitative and quantitative analysis.

The quantitative approach analyzes the potential failure modes through factors such as Reliability R(t), Unreliability F(t), Availability A(t), Unavailability *Q*(*t*) and Failure Rate λ(t). If we assume ‘*T*’ to be the random variable representing the time “*t*” to failure, then *R*(*t*) can be expressed as in Equation (3).

(3) R(t)=P(T≥t)

Conversely, *F*(*t*) can be defined as the probability of failure, before time “*t*”, as in Equation (4). As the occurrence of fault in a system is random by nature, by means of the law of probability, $\bar{R}\left(t\right)$ can be calculated as equal to Cumulative Distribution Function, i.e., $\bar{R}\left(t\right)=F_{fc}\left(t\right)$=1−R(t).

(4) $$F\left(t\right)=\bar{R}\left(t\right)=P\left(T<t\right)$$

In practice, it is more suitable to consider the probability associated with a small range of values, as the probability of “*T*” having an exact value of “*t*” will be significant. Hence, the probability density function *f*(*t*) is introduced, as shown in Equation (5),

(5) $$f\left(t\right)=\frac{dF\left(t\right)}{dt}=-\frac{dR\left(t\right)}{dt}$$

The probability of a component failing during a specific time period [x, x + ∆t], given that it is operating at time instant [*t* = x], is described as *λ*(*t*) and is closely associated with R(*t*), as in Equation (6),

(6) $$\lambda \left(t\right)=\frac{f\left(t\right)}{R\left(t\right)}$$

By integrating Equation (6), the expression for the reliability of a system can be derived as in Equation (7),

(7) $$R\left(t\right)=\exp(-\int_{0}^{t}\lambda \left(t\right)dt)$$

The FTA of the SED under research considers repeated events and the relationship between individual elements. Thus, *R*(*t*) is to be calculated as in Equation (8) for a series system, where “*R*_1_”, “*R*^2^” and “*R*_3_” represents the reliability of the sensing, processing and communication modules of the SED.

*R*(*t*) = *R*_1_(*t*) × *R*_2_(*t*) × *R*_3_(*t*) (8)

Qualitative analysis identifies the minimum component list that could cause a system to fail as a whole. The calculation of the minimal cut “*Ci*” set assumes that the components fail independently of one another, which is defined by Equation (9), where the individual events are represented as *X*_1_, *X*_2_, … *X_n_*.

(9) $$P\left(C_{i}\right)=\prod_{j-1}^{n}P\left(X_{j}\right)$$

In an FTA, each component’s significance must be individually identified to reduce the repair time; thus, Birnbaum’s measure *I^B^*(*i*|*t*), is used to assess a component’s significance, as defined in Equation (10),

(10) $$I^{B}\left(i|t\right)=\frac{\partial R\left(t\right)}{\partial R_{i}\left(t\right)}\;\mathrm{for}\;i=1,\;2,\;\ldots .\;N$$

**Figure 3:**
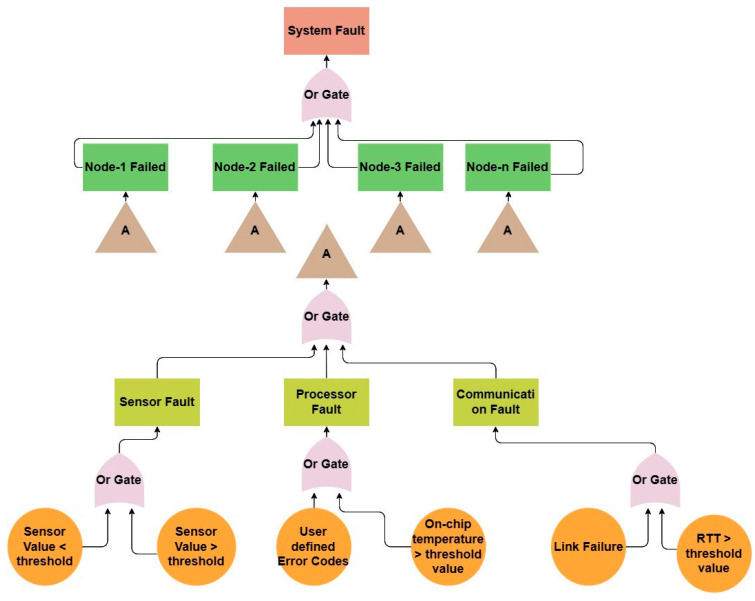
CH4-SED Fault Tree Architecture.

The FTA, as shown in Figure 3, is built to capture the CH4-SED’s typical operating environment in a programmatic approach. The top event denotes the overall system failure, and individual nodes comes next, where the base events represent each individual element’s failure.

#### 2.2.2. RF Classifiers

**Figure 4:**
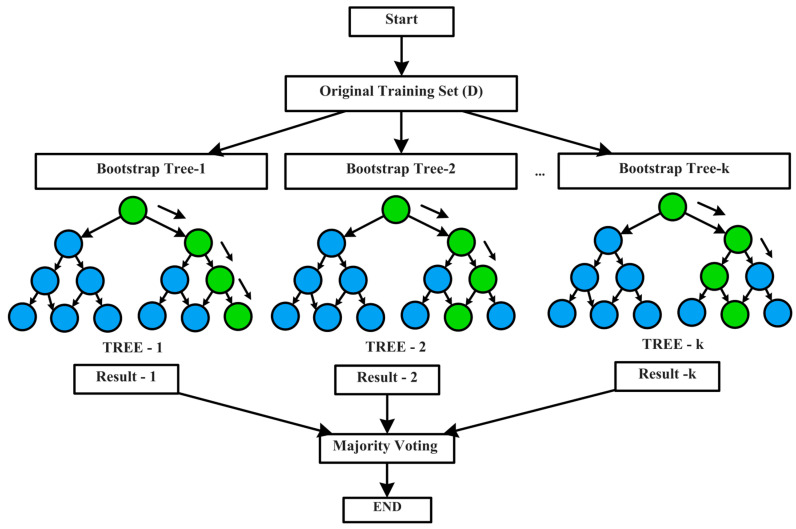
RF Architecture.

Recent attempts in sensor fault diagnosis have demonstrated the growing usage of RF, due to its benefit in dealing with small sample sizes and complex data formats [46]. RF is an ensemble algorithm that combines multiple weak decision trees grown from a randomly selected subspace, as illustrated in Figure 4. If there are “*n*” sample-points in a dataset “D” with “m” features, then the RF algorithm involves creating a forest of decision trees; each tree “h_k_” is individually constructed by recursively splitting the training data into smaller random subsets “F_k_”, based on input features “m”, until each subset contains only data points belonging to a single class. This is followed by the subspace randomization scheme, “D_k_” from “D” of size “*n*”, and the bagging scheme to resample with replacements. Once all the trees have been constructed, the algorithm produces the output “y” for the new input “x” using all of the decision trees. The final prediction is decided based on the majority voting scheme [47]. The decision tree node splits are chosen to decrease impurity at each step, which is governed by the Gini Index; it calculates how unclean or unequal the samples are, which are assigned to a node, based on a split at its parent, as represented in Equation (11).

(11) $$G=\sum_{i=1}^{C}p\left(i\right)*\left(i-p\left(i\right)\right)$$

### 2.3. Implementation and Working

**Figure 5:**
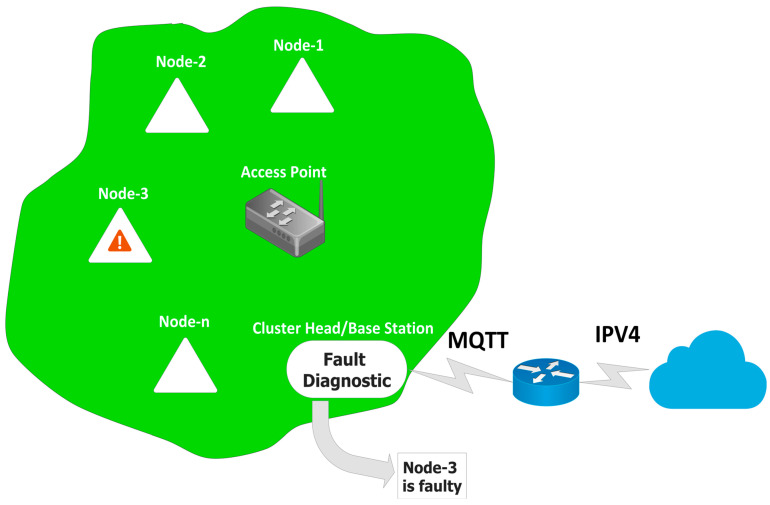
Illustration of the overall concept proposed.

The overall concept of the proposed algorithm is illustrated in Figure 5; when a sensing node is placed in an environment to collect the contextual information, they are prone to produce faults, due to physicality of logical fault. In an environment where the collected information is shared through the base-station or cluster head, an integrated algorithm can be utilized to scan the payload data before being sent to the Cloud [21]. This enables the identification of the fault at edge level, thus saving repair time, bandwidth consumption and energy, and also reducing stress on the Cloud [32]. The faults were induced manually as follows:

- Sensing Module
  ∘  The sensing device was exposed to various glass jars with different biogenic-process start dates to induce upper and lower threshold limit exceeding the faulted.
  ∘  Data line of the sensor was disconnected temporarily to induce no payload fault.
- Processor Module
  ∘  A forever-while (1) loop was used to induce the watchdog timer reset, resulting in the “E0” error.
  ∘  Programmatically, the threshold values were adjusted to be in lower values and the chip was exposed to a light source in order to induce an on-chip temperature error.
  ∘  A Regulated Power Supply system was used to induce a supply voltage error; error code “E1”.
  ∘  External load (resistor-bank) was added to consume more current to induce a short-circuit scenario; error code “E3”.
- Communication Module
  ∘  The ESP8266 was programmatically disconnected to induce link-failure; changing the SSID/password.
  ∘  The ESP8266 was connected to different access points with varied physical distance to induce RTT time-out.

**Figure 6:**
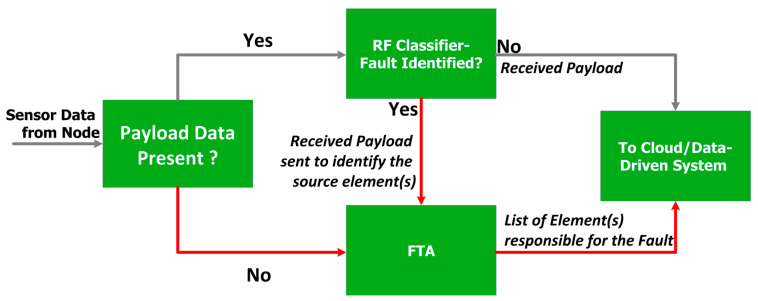
Block Diagram of the proposed integrated system.

The working of the proposed algorithm is illustrated in Figure 6 and the pseudocode of the proposed algorithm is given in Algorithm 1.

The sensor data is received and stored temporarily in a database and then checked for availability of payload content (“Sensor Value”, “Error Code”, “On-Chip Temperature”). If the payload content or data packet itself is missing, FTA function is called to test the communication link through Link-Failure and RTT tests. On the contrary, when payload content is present, the RF function is called by passing the payload content to test the faultiness of the senor data. Based on the output of the RF function, the payload is either passed to the Cloud or to the FTA function to identify the source of fault. The FTA function works based on the way it was invoked; RF function or Payload Checking function. If the FTA function has been invoked due to non-availability of payload, and if the links are able to be established and RTT values are within the threshold value, then the processor or sensor is identified to be the faulty element; if not, the communication module is concluded to be faulty. If the FTA function has been invoked from the RF function, then Sensor fault and processor fault tests are carried out as follows; the received payload data is verified against the pre-defined threshold values to identify the element(s) responsible for the fault. The approach is detailed in Algorithm 1.
**Algorithm 1:** Integrated Fault Detection and Identification ApproachFunction ProcessSensorData:  Input: Excel file path  data ← ReadExcelData(excelFilePath)//Read sensor data from Excel  if data is present then    RFFunction(data)//Call RF function with sensor data  else    FTAFunction()//Call FTA function Function RFFunction(data):  result ← Trained Random Forest model(data)//Check for faulty data  if result is not faulty then    SendToCloud(data)//Send data to cloud  else    FTAFunction()//Call FTA function Function FTAFunction():  if sensor data is present then    SensorFault()//Call SensorFault function    ProcessorFault()//Call ProcessorFault function  else    LinkFailure()//Call LinkFailure function    RTTFunction()//Call RTTFunction Function SensorFault():  //Paraphrase the sensor value from the payload and check against threshold value  … Function ProcessorFault():  //Paraphrase the processor value/Error codes from the payload and check against threshold value  … Function LinkFailure():  //Base station tries to ping the particular node  … Function RTTFunction():  //A RTT measurement is done and cross-checked against the threshold value  … Function SendToCloud(data):  //The sensor value or the list of faulty equipment is sent to the Cloud through MQTT protocol.  …

## 3. Results and Discussions

**Figure 7:**
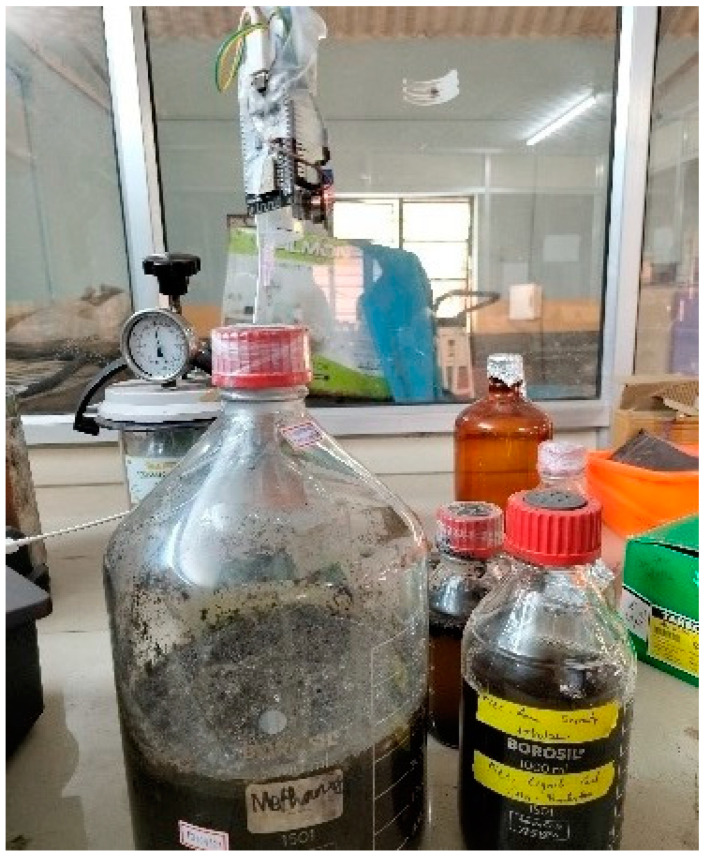
Sensing CH4 in a controlled environment.

To assess the effectiveness of the proposed integrated algorithm, a real-time, CH4-sensing SED was utilized, as shown in Figure 7. From the institute’s Bio-plant, where biogenic CH4 is produced from coal waste washeries, the dataset was generated.

### 3.1. Datasets

The data were gathered in-house from a MQ4 sensor fixed to a biogenic methane production unit. The readings were noted for 10 min continuously without fault, followed by adjusting different units, such as the sensor, microcontroller and Wi-Fi module to induce faults. The fault induced data were collected for ten minutes each, respectively. In total, 1515 datapoints were collected under five sets of features (“Sensor Value (PPM)”, “Supply Voltage (V)”, “Overall Circuit Current (mA)”, “On-Chip Temperature (°C)”, “Error Code”), where overall-voltage and circuit current were used for error code deduction, and the target column was labeled as “working” and “faulty”. Table 1 shows the sample of the data collected; as the dataset was generated in-house, it was imbalanced—1010 faulty and 405 non-faulty samples. The full dataset was randomly split into 70% (1061 samples) for training and 30% (454 samples) for testing.

The RF and FTA models were simulated using Python-3 language in the Jupyter Notebook through the Anaconda environment on a Windows platform with an Intel (R) Core (TM) i7 CPU @3.40 GHz and 16 GB of RAM-memory. For programming ML models, the Scikit-learn library was used, and the parameters utilized to design the models are as follows: RF model—number of estimators = 50, max depth = 4, max features = ‘sqrt’; KNN model—number of neighbors = 5; SVM model—kernel = ‘poly’, C = 0.4, and random state = 20. The following section details the simulation and implementation outcomes.

### 3.2. Performance of RF Classifier

**Figure 8:**
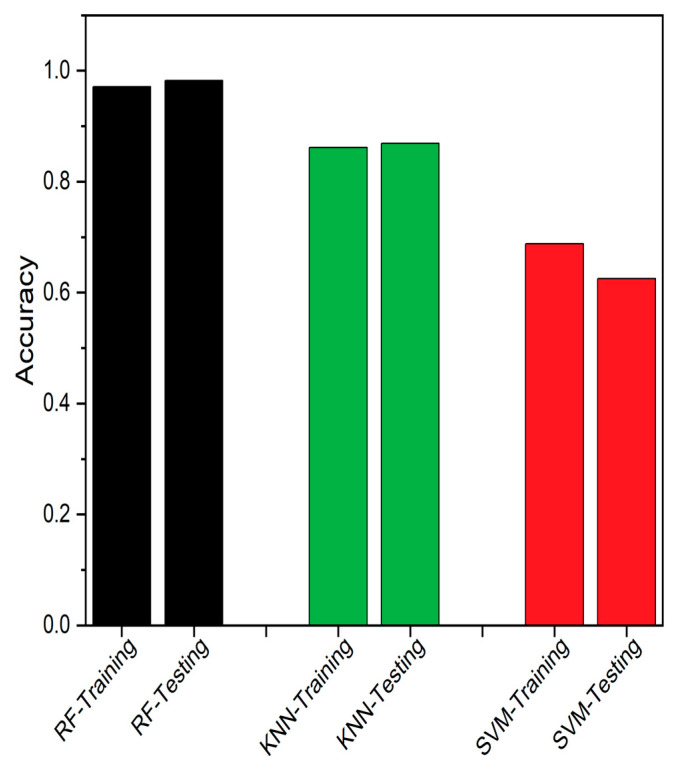
Accuracy comparison between RF, KNN and SVM.

Four different measures were used to assess the performance of the proposed algorithm. Accuracy [35] was the first metric used, as defined in Equation (12). The comparative outcomes between the classifiers are shown in Figure 8.

(12) $$A=\frac{\mathrm{TP}+\mathrm{TN}}{\mathrm{TP}+\mathrm{TN}+\mathrm{FP}+\mathrm{FN}}\%$$

**Figure 9:**
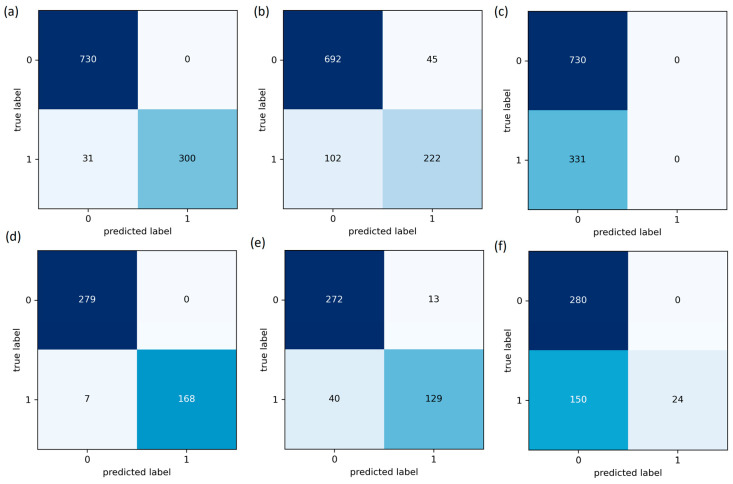
Confusion Matrix of (**a**) RF-Training, (**b**) KNN-Training, (**c**) SVM-Training, (**d**) RF-Testing, (**e**) KNN-Testing and (**f**) SVM-Testing.

**Figure 10:**
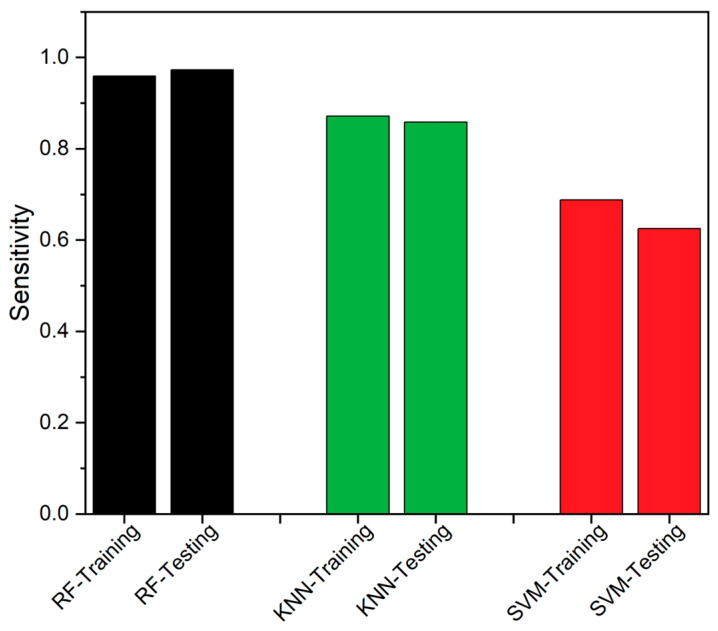
True Positive Rate comparison between RF, KNN and SVM.

The True Positive Rate (TPR), also known as sensitivity [48], was used as the second metric, which was calculated as defined in Equation (13), where “TP” represents True Positive and “FN” represents False Negatives. Figure 9 presents a comparison of the confusion matrix for RF, KNN, and SVM, and Figure 10 shows the TPR comparison between the classifiers.

(13) $$\mathrm{TPR}=\frac{\mathrm{TP}}{\mathrm{TP}+\mathrm{FN}}$$

**Figure 11:**
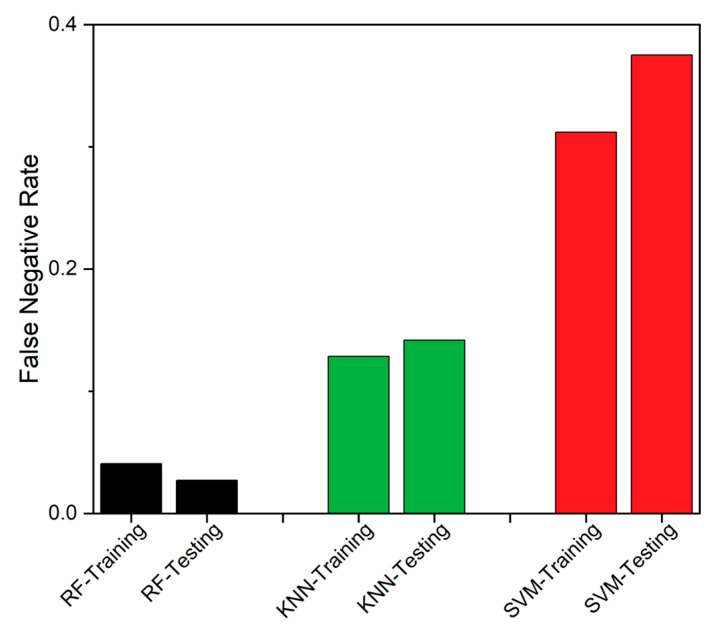
False Negative Rate comparison between RF, KNN and SVM.

The third metric used is MCC [49], which ranks fault diagnosis according to accuracy, as in Equation (14), where the False Positive (FP) was descripted as a number of wrongly labeled faulty data. The MCC ranges the algorithms between −1 to +1, and the algorithm that is nearer to +1 indicates a strong correlation between the test and reality. Figure 11 represents the comparison between all the classifiers used; on the basis of their False Negative Rate (FNR) and the performance variation of each algorithm being clearly projected by the bars.

Table 2 lists the order of all the classifiers. In comparison to all of the classifiers, RF is seen to outperform others. RF is seen to be closest to +1, thus rated as the best classifier, and KNN was rated as the second most compatible method.

(14) $$\mathrm{MCC}=\frac{\left(\mathrm{TP}*\mathrm{TN}\right)-\left(\mathrm{FP}*\mathrm{FN}\right)}{\sqrt{\left(\mathrm{TP}+\mathrm{FP}\right)\left(\mathrm{TP}+\mathrm{FN}\right)\left(\mathrm{TN}+\mathrm{FP}\right)\left(\mathrm{TN}+\mathrm{FN}\right)}}$$

**Figure 12:**
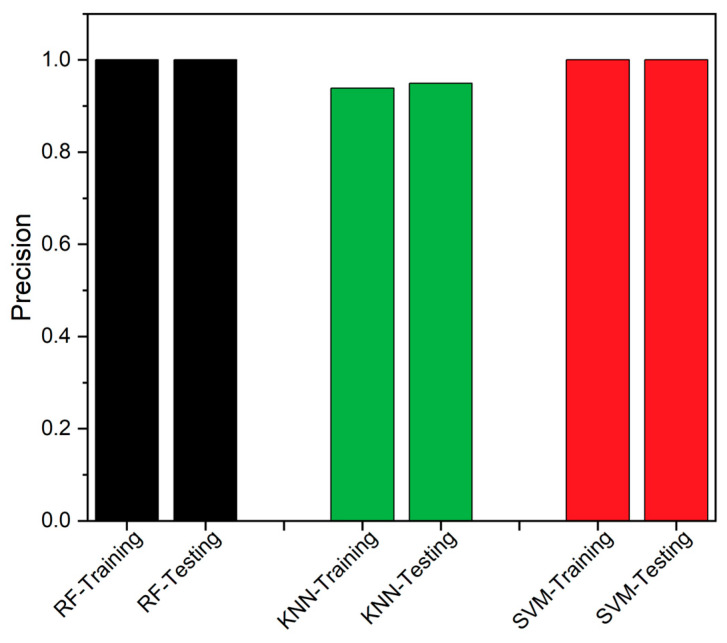
Precision—Comparison between RF, KNN and SVM.

**Figure 13:**
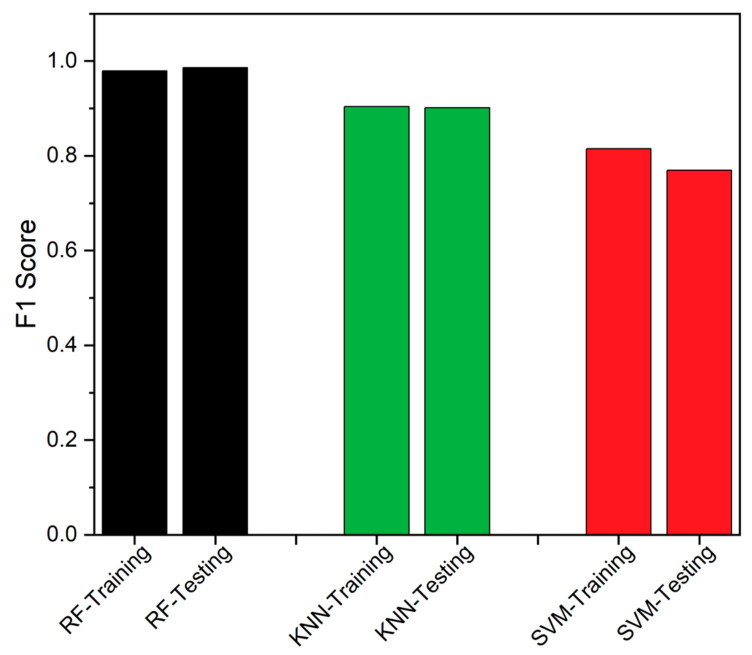
F1-score comparison between RF, KNN and SVM.

The F1 score is the fourth metric, utilized to assess performance. On the basis of FN and FP, it serves as a statistical metric to evaluate the effectiveness of a specific classifier [50,51]. It is defined as the harmonic mean of recall and precision, as defined in Equation (15). The Precision value is described as the correctness of judgement and the values obtained in the current study are projected in Figure 12. In Figure 13, the F1-score of all the classifiers are compared, and it is inferred that RF has the highest value, showcasing its better performance.

(15) $$F1_{\mathrm{score}}=\frac{2*\;\mathrm{Precision}*\;\mathrm{Recall}}{\mathrm{Precision}+\;\mathrm{Recall}}$$

### 3.3. Performance of the FTA

The FTA was programmatically implemented as a function based on “if-then” rules. The conditions were implemented using logical operators. The performance of FTA was measured using execution time as the metric, whose results are noted in row three of Table 3. From the table, it is understandable and validates our claim; FTA’s execution time increases with the number of sensor data. Based on the iterations of the test carried out, the following observations have been noted. All three elements are identified to be a minimal cut set element. Also, the reliability of the sensor element was identified to be poor compared to the processor and communication module, but identified to be the significant component in SED.

### 3.4. Performance of the Integrated Algorithm

The total time required to identify the fault using the FTA model is represented as “T_fta_”; the time required to identify the fault using a classifier model, such as RF, KNN and SVM, is represented as “T_rf_”, “T_knn_”, and “T_svm_”, respectively. The percentage of reduced execution time is calculated based on Equation (16), where the time taken by the integrated model is calculated as the sum of time taken by a classifier model multiplied by the number of datapoints, plus time taken by the FTA model, multiplied by the number of known faults:

(16) $$\mathrm{Reduced}\;\mathrm{Execution}\;\mathrm{Time}=\frac{\left(\mathrm{Time}\;\mathrm{taken}\;\mathrm{by}\;\mathrm{FTA}\;\mathrm{model}\right)-\left(\mathrm{Time}\;\mathrm{Taken}\;\mathrm{by}\;\mathrm{Integrated}\;\mathrm{Model}\right)}{\mathrm{Time}\;\mathrm{taken}\;\mathrm{by}\;\mathrm{FTA}\;\mathrm{model}}\%$$

A set of 158 sample datapoints with 37 known faults were used to test the integrated algorithm. The T_fta_ was calculated to be 42.65 microseconds. On the other hand, T_rf_ was calculated as 20.96 ms, T_knn_ was calculated as 25.30 ms and T_svm_ was calculated to be 22.66 ms. Thus, for a set of 158 datapoints, the total time required by FTA was 6.73 ms, whereas the time required by RF-FTA analysis was 3.82 ms (RF _(158)_ + FTA _(37)_). This is an 27.73% decrease in executional time requirement compared to a traditional FTA approach. Table 3 illustrates the different iterations performed with known datapoints and faults in them, where row four indicates the percentage of time saved on employing the integrated RF-FTA approach.

From the table, it can be inferred that the proposed system consumes less time to scan the datapoints for fault, in comparison to the static time value, in the case of a more traditional FTA approach. Also, its inferred that the SVM model follows a close execution time requirement next to RF, whereas KNN, although producing better results in terms of other performance metrics, only performed next to RF and SVM in terms of execution time. In addition, it is inferred that the number of faulty datapoints increases the overall execution time, irrespective of the classifier model that was used. Thus, the proposed edge fault detection and identification algorithm has advantages over traditional methods, such as:

- Reduced latency: Edge fault detection algorithms can detect faults much faster than traditional methods, which can help to prevent costly downtime.
- Improved accuracy: Machine learning algorithms can learn to identify patterns in data, which can lead to improved accuracy in fault detection.

## 4. Conclusions

SEDs are prone to produce erroneous values due to various factors, and even a modest undetected fault could result in system fail. These faults can affect the SED’s availability and impact the overall data-driven system. The traditional FTA is the better solution to detect and identify the fault, but consumes higher executional time, which is likely to increase with additional problematic events. Thus, an edge deployable fault detection and identification algorithm is proposed, as discussed in Section 2, by integrating RF and FTA. The RF classifier is employed to detect the fault, while the FTA is utilized to identify the source of the fault. We used data from a healthy CH4 sensing node, which was injected with different forms of faults, as discussed in Section 2, to assess the proposed model’s performance. The proposed integrated algorithm provides better algorithm-complexity and accuracy when compared to a standalone FTA or standalone classifiers, such as RF, SVM and KNN, as discussed in Section 3. The performance of the classifiers was assessed using accuracy, TPR, MCC, Precision, FNR and F1-score, as performance metrics. From the field-experiment, RF produced 97.27% accuracy and outperformed both SVM and KNN. Also, the suggested methodology’s experimental findings demonstrated to work well, which is evident from Table 3 and Figure 8, Figure 9, Figure 10, Figure 11, Figure 12 and Figure 13. A 27.73% reduced time-consumption was achieved for 158 sample datapoints with 37 known faults, with correct fault-source and less computational resource, in comparison with a traditional FTA approach. Regarding future research, different algorithms can be experimented to invoke FTA and further reduce the execution time or modify SVM to have a better trade-off compared to the current method used.

In the current study, only one node has been used to verify the integrated approach. Also, the cluster head has been orchestrated in a personal computer setup to replicate the edge environment. In the future, modifications to the overall algorithm to incorporate multiple nodes and implement the algorithm in real hardware, such as a Raspberry Pi, is required to further assess the prosperity of the proposed approach.

## Figures and Tables

**Table 1:** Structure of data collected.

Sensor Value (PPM)	Battery Voltage (V)	Overall Circuit Current (mA)	On-Chip Temperature (°C)	User Defined Error Code	Label
325	3.2	199	37.9		Working
245	3	201	34.3	E0	Faulty
312	3.1	200	38.3		Working
212	2.9	198	33.1	E1	Faulty
341	3.3	202	39.8		Working
222	2.8	198	33.5	E3	Faulty
265	3	200	36.3		Working
305	3.2	203	38		Working

**Table 2:** Comparison of various performance measures of RF, SVM & KNN.

Measure	Training			Testing		
	RF	KNN	SVM	RF	KNN	SVM
Sensitivity	0.9593	0.8715	0.688	0.9728	0.8582	0.625
Specificity	1	0.8315	-	1	0.8943	-
Precision	1	0.9389	1	1	0.949	1
Negative Predictive Value	0.9063	0.6852	0	0.9524	0.7333	0
False Positive Rate	0	0.1685	-	0	0.1057	-
False Discovery Rate	0	0.0611	0	0	0.051	0
False Negative Rate	0.0407	0.1285	0.312	0.0272	0.1418	0.375
Accuracy	0.9708	0.8615	0.688	0.9824	0.8691	0.625
F1 Score	0.9792	0.904	0.8152	0.9862	0.9013	0.7692
Matthews Correlation Coefficient	0.9324	0.6624	-	0.9625	0.7166	-

**Table 3:** Time saved on employing the integrated approach- Different iterations.

Description	Iteration-1	Iteration-2	Iteration-3	Iteration-4	Iteration-5	Iteration-6
No. of Sample Datapoints	50	63	72	80	95	158
No. of known fault	10	11	10	8	12	37
Time (ms)—FTA	2.13	2.69	3.07	3.41	4.05	6.74
Time (ms)—RF-FTA	1.47	1.79	1.94	2.02	2.50	4.89
Time (ms)—KNN-FTA	1.69	2.06	2.25	2.37	2.92	5.58
Time (ms)—SVM-FTA	1.56	1.90	2.06	2.15	2.66	5.16
Percentage reduction execution time between FTA vs. RF-FTA	30.86%	33.40%	36.97%	40.86%	38.22%	27.73%

## Data Availability

The data presented in this study are available through email upon request to the corresponding author.
